# A new species of *Onitis* Fabricius, 1798 from south-eastern Africa (Coleoptera, Scarabaeidae, Scarabaeinae, Onitini)

**DOI:** 10.3897/zookeys.900.39284

**Published:** 2019-12-31

**Authors:** François Génier, Philippe Moretto

**Affiliations:** 1 Beaty Centre for Species Discovery, Canadian Museum of Nature, P.O. Box 3443, Station D, Ottawa, Ontario, K1P 6P4 Canada Canadian Museum of Nature Ottawa Canada; 2 2, rue Marcel Sambat, 83200 Toulon, France Association Catharsius Toulon France

**Keywords:** Democratic Republic of the Congo, Dung beetle, Mozambique, new species, *Onitis albertcollarti*, Tanzania, Zambia

## Abstract

*Onitis albertcollarti***sp. nov.** is described and illustrated. The new species is closely related to and occurs sympatrically with *O. lycophron* Klug, 1855.

## Introduction

While surveying dung beetles in Zambia and Mozambique, we found a series of specimens that key out to *Onitis lycophron* Klug, 1855 in the Ferreira (1978) key to the Afrotropical species of Onitini. These specimens were clearly distinct from *O. lycophron* and all other *Onitis* based on a unique protibial protrusion in males and the distinct fine rugae on the apical surface of the parameres and belonged to a new species. This new species belongs to the *O. lycophron* species complex (defined by Krikken 1977), which is part of the larger *Onitis* 19^th^ species group as defined by Janssens (1937).

The main purpose of describing this new species of *Onitis* is to make the name available for a field guide of the dung beetle species of Parque Nacional das Quirimbas (FG, in preparation). Although the taxonomy of the species belonging to group 19 needs to be reviewed, the uniquely shaped parameres unambiguously separate this undescribed taxon from all others in the genus.

## Materials and methods

Type specimens were deposited in the following collections:

**CMNC** Canadian Museum of Nature, Ottawa, Ontario, Canada;

**FGIC** François Génier personal collection, Gatineau, Québec, Canada;

**IRSNB** Royal Belgian Institute of Natural Sciences, Brussel, Belgium;

**JFJC** Jean-François Josso personal collection, Muzillac, France;

**PMOC** Philippe Moretto personal collection, Toulon, France;

**RMIC** Collection Robert Minetti, La Ciotat, France.

The methods are the same as described in Génier and Moretto (2017). All specimens are types and have a World Scarab. Database number.

## Taxonomy

### Onitis albertcollarti sp. nov.

Taxon classificationAnimaliaColeopteraScarabaeidae

35EBBF64-7B88-53B4-8D02-51F343CA201C

http://zoobank.org/560344F2-06A1-4BD2-B5BD-ABF1C2748DC1

Figures 1–9

#### Type locality.

1 km SE Kacheleko Outpost (15°01'35"S 26°25'23"E, 1250 m), Central Province, Zambia.

***Holotype*** ♂ (CMNC, WSD00038914): [ZAMBIA: CENTRAL PR. | 1 km SE Kacheleko Outpost, | 1250 m, 15°01'35"S 26°25'23"E | 10.XII.2010, Central Zambezian | & Miombo woodland, prairie edge | light trap, F. Génier, 2010-56] ; [WORLD | SCARAB. | DATABASE | WSD00038914] barcode label; [HOLOTYPE ♂ | *Onitis* | *albertcollarti* | des. Génier & Moretto, 2019] red card.

***Allotype*** ♀ (CMNC, WSD00038918): same data as holotype.

***Paratypes*** 68 ♂♂, 55 ♀♀, 402 unsexed specimens. Democratic republic of the Congo: KATANGA, [unspecified locality], [no date], [anonymous], – 1 ♂ (IRSNB); Mozambique: CABO DELGADO, Ravia (site 1), P.N. Quirimbas, 380 m (12°39'41"S, 39°25'22"E), 1.i.2013, F. & S. Génier & M. Denja, eastern Miombo woodlands, light trap (2013-24) – 1 ♀, 1 ♂ (FGIC); same locality, 2.i.2013, F. & S. Génier & M. Denja, eastern Miombo woodlands, light trap (2013-26) – 1 ♂ (FGIC); Ravia (site 6), P.N. Quirimbas, 380 m (12°31'2"S, 39°18'38"E), 3.i.2013, F. & S. Génier & M. Denja, eastern Miombo woodlands, light trap (2013-35) – 2 ♂♂ (FGIC); Taratibu (site 4), P.N. Quirimbas, 340 m (12°47'32"S, 39°41'50"E), 7.i.2013, F. & S. Génier & M. Denja, eastern Miombo woodlands, elephant dung (2013-39) – 3 ♂♂ (FGIC); Taratibu (site 5), P.N. Quirimbas, 340 m (12°47'3"S, 39°42'10"E), 10.i.2013, F. & S. Génier, eastern Miombo woodlands, elephant dung (2013-67) – 1 ♀, 3 ♂♂ (FGIC); Tanzania: DODOMA, Dodoma, (6°11'S, 35°46'E), xii.2006, [anonymous], – 43 specimens (RMIC); IRINGA, Tandala, Ruaha National Park, 912 m (7°47.412'S, 35°30.219'E), 6.xii.2006, R. Minetti, – 4 ♀♀, 4 ♂♂, 96 specimens (FGIC, PMOC); MOROGORO, savane de Mikesse Hills, 378 m (6°14.457'S, 37°58.312'E), 12–14.xii.2006, R. Minetti, – 1 ♂ (PMOC); Uluguru Mountains, (7°6'S, 37°39'E), xii.2006, Local collectors, – 103 specimens (PMOC); Vuma Hills, (7°25'S, 37°8'E), 3–5.i.2009, D.C. Moore, – 3 ♀♀, 1 ♂ (PMOC); RUKWA, 10 km N Namanyere, Luafi Game reserve, 510 m (7°27.289'S, 30°54.498'E), 19.xi.2006, R. Minetti, – 1 ♂ (PMOC); Zambia: CENTRAL PROVINCE, 1 km SE Kacheleko Outpost, 1250 m (15°1'35"S, 26°25'23"E), 10.xii.2010, F. Génier, central Zambezian & Miombo woodland, prairie edge, light trap (2010-56) – 1 ♀, 3 ♂♂ (CMNC, FGIC); 25 km NE Lilemone, 1250 m (15°13'14"S, 26°19'41"E), 5.xii.2010, F. Génier, central Zambezian & Miombo woodland, light trap (2010-46) – 6 ♀♀, 8 ♂♂ (FGIC); same locality, 6.xii.2010, F. Génier, central Zambezian & Miombo woodland, dung trap (2010-47) – 1 ♂ (FGIC); 5.6 km SW Kacheleko Outpost, 1250 m (15°3'28"S, 26°23'55"E), 7.xii.2010, F. Génier, central Zambezian & Miombo woodland, prairie edge, dung trap (2010-48) – 7 ♀♀, 7 ♂♂ (FGIC); 6.2 km W Mukambi Lodge Jct. on M9, 1100 m (14°57'2"S, 25°56'21"E), 18.xi.2010, F. Génier, open central Zambezian & Miombo woodland, elephant dung (2010-02) – 4 ♀♀, 3 ♂♂ (FGIC); 6.5 km N Chunga, 1100 m (14°59'40"S, 26°1'11"E), 4.xii.2010, F. Génier, open central Zambezian & Miombo woodland, light trap (2010-43) – 15 ♀♀, 18 ♂♂ (FGIC); Chunga, Kafue National Park [site 1], (15°2.362'S, 25°59.437'E), 11–12.xii.2009, Josso, Juhel & Minetti, piège lumineux – 20 specimens, 1 ♀, 1 ♂ (JFJC); Kacheleko Wildlife Outpost, Kafue National Park, (15°1'S, 26°25'E), 2–3.xii.2007, Josso, Juhel & Monfort, piège lumineux – 6 ♀♀, 4 ♂♂ (JFJC); same locality, 6–7.xii.2008, J.-F. Josso & R. Minetti, piège lumineux – 2 ♀♀ (JFJC); same locality, 10–18.xii.2009, Josso, Juhel & Minetti, piège lumineux – 91 specimens (JFJC); Kafue river east, (14°57'S, 25°55'E), 4.xii.2007, Josso, Juhel & Monfort, – 49 specimens (JFJC); Mukambi Safari Lodge, 1250 m (14°58'32"S, 25°59'29"E), 8.xii.2010, F. Génier, open central Zambezian & Miombo woodland, light trap (2010-50) – 2 ♀♀, 1 ♂ (FGIC); same locality, 9.xii.2010, F. Génier, open central Zambezian & Miombo woodland, light trap (2010-52) – 1 ♀, 1 ♂ (FGIC); COPPERBELT, Kasompe, (12°36'S, 27°53'30"E), ii.1982, [anonymous], – 1 ♀, 3 ♂♂ (PMOC).

#### Diagnosis.

Male *Onitis albertcollarti* sp. nov. will key to couplet 4 on page 159 in the Krikken (1977) key to species of the *O. lycophron* species complex. The presence of a single tooth on the profemur ventral surface on basal half (Fig. 3, arrow) will separate it from *O. mendax* Gillet, 1918 (interrupted carina) and *O. pseudojanssenii* Krikken, 1977 and *O. janssenii* Gomes Alves, 1854 (both species with two separate protibial protrusions on the ventral surface basally in moderate to large males).

**Figures 1–9.**
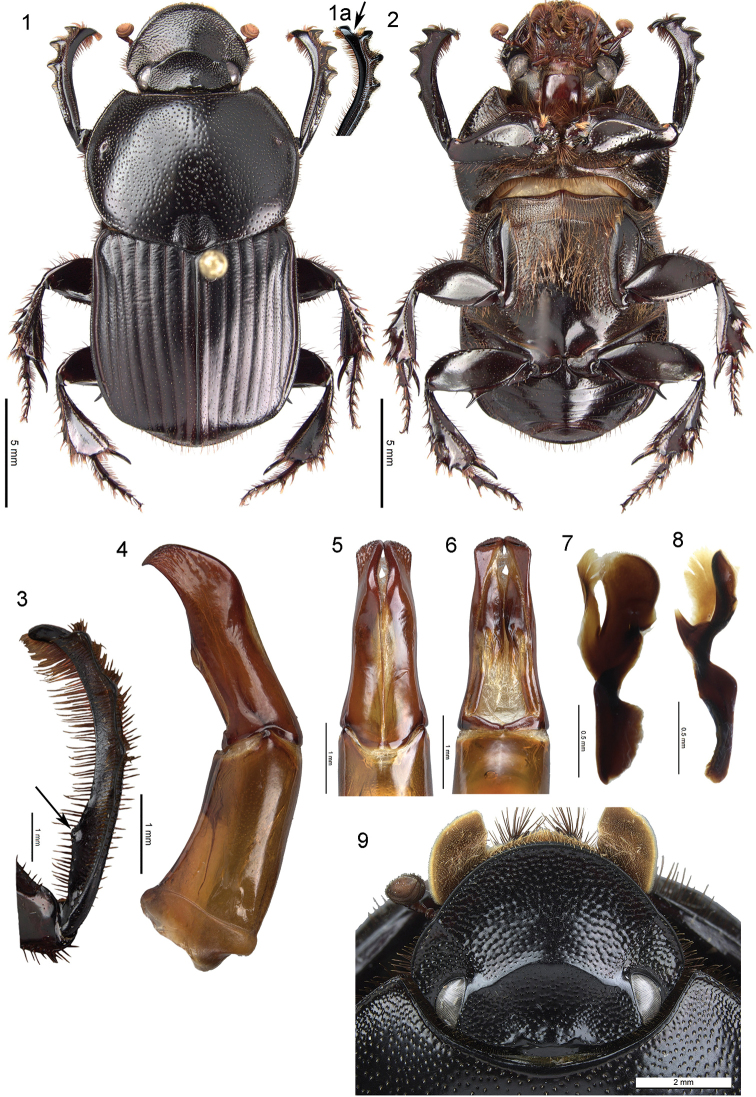
*Onitis albertcollarti* sp. nov. **1–8** male holotype **9** female allotype **1** habitus, dorsal view **1a** protibia, slightly oblique dorsal view to show apical tooth shape **2** habitus, ventral view **3** protibia, oblique view **4** aedeagus, lateral view **5** parameres, dorsal view **6** parameres, ventral view **7** frontolateral peripheral endophallite (FLP), flat view **8** frontolateral peripheral endophallite (FLP), side view **9** head, dorsal view.

*Onitis albertcollarti* will key to *O. lycophron* (couplet 12, page 323) in the Ferreira (1978) key to Onitini species. Males differ from *O. lycophrons* by the distinct projecting apical tooth of the protibia (Fig. 1a, arrow) and the external edges of the apical tooth forming a distinct angle with the anterior edge of the apical lateral tooth. Females differ from *O. lycophron* by the smaller subtriangular pygidium.

#### Description.

Holotype ♂ (Figs 1–8). ***Overall aspect*.** Length 20.0 mm, maximum width 9.5 mm. Colour dark brown to black, lacking metallic sheen. Dorsal surface slightly sericeous on head and pronotum, elytra less glossy. Setae minute on head, pronotum, and dorsal surface of elytra, with longer setae on elytral apical declivity. Venter with long dense pubescence on mesosternum and median lobe of metasternum. ***Head*.** Anterior clypeal edge rounded, margin abruptly upturned. Clypeogenal sutures not carinate, weakly defined. Clypeal surface with short, transversal scabrous punctures. Genal surface finely granulate. Clypeofrontal carina arcuate medially, slightly sinuous and tuberculate near the clypeogenal suture. Frontal surface finely punctate medially, with denser granulate punctures laterally. Frontal tubercle obtuse, in line with cephalic posterior edge. ***Pronotum*.** Lateral edges broadly arcuate in dorsal view, maximum width midway between anterior angle and posterior angle. Surface moderately convex. Pronotal punctures simple and coarse, becoming scabrous on anterior angles; punctures irregularly distributed, separated by one to six diameters. Posteromedian fossae fused, surface with coarse microsculpture and with fine, irregular, scabrous punctures. Posterior edge finely crenulate on each side of posteromedian fossae. ***Scutellum*.** Small and triangular. ***Elytra*.** Moderately convex. Striae 1–7 moderately wide, slightly wider at basal third. Strial punctures weakly defined on apical declivity. Interstriae 1–8 feebly convex, with fine, irregularly-spaced punctures. Elytral striae 8 straight, wide, and deeply impressed from humeral callus to junction with striae 7. Elytral interstria 9 narrowly bulging dorsally. Elytral striae 8 and 9 wide and deeply impressed at apical third. ***Pygidium*.** Subtriangular, distinctly smaller than in *O. lycophron*. Pygidial surface convex, sericeous, with scattered, minute, setigerous granules. ***Antennal club*.** Fulvous, mostly covered with dense, minute, yellow setae and some scattered long, brown setae. ***Ventrites*.** Metasternal surface with two more-or-less triangular depressions on each side of midline posteriorly. Abdominal sternite 3 with few minute, scattered setae medially. ***Legs*.** Profemur anterior surface flat with minute punctures. Apicoventral edge produced into a small, inwardly bent denticle. Protibia long and slender, anterior half evenly bent inward. External edge with four teeth. Apex produced into a semi-trapezoidal and downwardly bent tooth flanked internally by a short but thick setal brush (Fig. 1a, arrow). Distal edge of apical tooth forming a distinct angle with external edge of apical projection. Longitudinal carina of ventral surface obtusely toothed at basal fourth (Fig. 3, arrow). Mesofemur posterior edge crenulate in ventral view, lacking a denticle or projection on apicoventral angle. Mesotibial internal edge straight. Metafemur lacking ventrally bent tooth on anterior edge at middle, posterior edge produced into an obliquely oriented, acute tooth medially, basal portion of tooth never fused with ventroposterior edge of metafemur. ***Aedeagus*.** Phallobase as long as parameres (Fig. 4). Parameres with apical surface covered with coarse, fused granulation forming fine longitudinal rugae (Figs 4, 5). Frontolateral peripheral endophallite (FLP) bilobate, with a spiniform process at distal third (Figs 7, 8).

#### Variation.

Measurements (59 ♂♂, 46 ♀♀). Length: male 15.0–21.0 mm (18.0 ± 1.5 mm), female 15.5–21.0 mm (18.1 ± 1.3 mm). Female as male except clypeal edges ogival in dorsal view (Fig. 9); pygidium smaller and triangular with surface flat and with denser, minute, setigerous granules; legs unmodified, except for mesofemoral posterior edge as in male. Some variation in the coarseness of dorsal microsculpture with some individuals appearing entirely dull.

#### Etymology.

While visiting the Royal Belgian Institute of Natural Sciences in Brussels, we found a specimen of this species bearing the “*Onitis collarti*” paratype label of André Janssens. Since Janssens’ name was never formally described we decided to honour this homage with the modification to “*O. albertcollarti*”. Albert DCH Collart was a colleague and friend of André Janssens a well-known scarab worker. Collart first worked as a sanitary agent in the former Belgian Congo from 1923 to 1930. For health reason he had to come back to Belgium and started to work as a scientific collaborator for the entomology department of the former Royal Museum of Natural History in 1932. He was promoted several times and concluded his career as the Laboratory Director of the Institution in 1964. He retired in January 1965 and remained associated as a scientific collaborator of the Royal Belgian Institute of Natural Sciences until his death in 1993.

#### Distribution.

From southern Democratic Republic of the Congo (Katanga) to Tanzania in the north and eastern Zambia through northern Mozambique in the south. *Onitis albertcollarti* occurs sympatrically with *O. lycophron* on most of its northern distribution.

#### Natural history.

Specimens with data were collected using pitfall traps baited with human faeces and elephant dung and were attracted to light traps. Some individuals were collected in eastern Miombo woodlands and central Zambezian woodlands.

## Supplementary Material

XML Treatment for Onitis albertcollarti
